# Sepsis Bundle Compliance Before and After the Implementation of a Protocolized Sepsis Program

**DOI:** 10.7759/cureus.96663

**Published:** 2025-11-12

**Authors:** Rebecca Valean, Jenna Holzhausen, Erika Waldsmith, Lisa Hall Zimmerman

**Affiliations:** 1 Department of Pharmaceutical Services, William Beaumont University Hospital, Royal Oak, USA

**Keywords:** antibiotic usage, fluid resuscitation, sepsis and shock physiology, sepsis bundles, severe sepsis

## Abstract

Introduction

Protocolized approaches to sepsis management, or “sepsis bundles,” have been increasingly adopted by medical institutions to facilitate prompt treatment and improve outcomes. This study aimed to evaluate the effects of a protocolized sepsis algorithm to improve Centers for Medicare & Medicaid Services (CMS) hour-3 bundle compliance.

Methods

This retrospective, single-center study included patients aged≥ 18 years admitted for severe sepsis or septic shock who received at least one dose of antibiotics within 24 hours of admission, either Pre-Code Sepsis (PC) between 3/15/22 and 6/15/22, or After-Code Sepsis implementation (AC) between 3/15/23 and 6/15/23. The Code Sepsis program consists of a team-based algorithm of sepsis bundle components, including but not limited to clinical assessments, fluids, antibiotics, and laboratory studies, to enhance their timely completion in the emergency department. The primary outcome evaluated hour-3 bundle compliance. Secondary outcomes included hour-1 and hour-6 bundle compliance. Data were analyzed using SPSS Statistics v.29.0 (IBM Corp., Armonk, NY).

Results

A total of 118 patients were included in the study (59 PC vs. 59 AC). The mean patient age was 63 ±18 years; 45% were male, and 42% were diagnosed with septic shock. Hour-3 bundle compliance trended toward improvement (25% PC vs. 41% AC, p=0.07) and similarly observed for the hour-6 bundle (51% PC vs. 64% AC, p=0.13). Hour-1 bundle compliance significantly improved (10% PC vs. 24% AC, p=0.03). Hospital length of stay was significantly shorter after Code Sepsis (eight (0.7-50) days PC vs. five (0.4-34) days AC, p=0.005). No difference between in-hospital all-cause mortality (20% PC vs. 10% AC, p=0.13) or hospice disposition (8% PC vs. 2% AC, p=0.20) was observed.

Conclusions

Code Sepsis implementation was associated with a trend toward improved hour-3 and hour-6 bundle compliance, significantly improved hour-1 bundle compliance, and reduced hospital length of stay. Further prospective studies of protocolized approaches to sepsis management are required to determine the impact on mortality.

## Introduction

Sepsis is a medical emergency described as life-threatening organ dysfunction caused by the body’s dysregulated response in the setting of infection [1]. Despite advances in resuscitative care, sepsis remains a leading cause of morbidity and mortality worldwide, affecting millions each year. Based on data from the Centers for Disease Control and Prevention, at least 1.7 million adults in the U.S. develop sepsis annually, and at least 350,000 adults who develop sepsis die during their hospitalization or are discharged to hospice [2]. The substantial mortality burden of sepsis has been well observed, with emerging data also suggesting a time-sensitive increase in the risk of mortality without prompt intervention. For instance, a 9% increased mortality risk for each additional hour between an emergency department presentation and antibiotic administration has been previously described [3]. As such, international guidelines and institutions have increasingly adopted systematic approaches to enhance the timely identification and management of sepsis. This is most notably pioneered by the Surviving Sepsis Campaign (SSC), which launched initiatives to reduce the sepsis mortality burden, including the outlining of sepsis “bundles” since the first SSC guidelines were published in 2004 [4].

While these bundles have undergone various revisions with recent guideline updates, they continue to outline core elements of resuscitation, which are to be completed in the initial hours of a patient presenting with sepsis. The hour-1 and hour-3 bundles include core components of resuscitation, such as initiation of crystalloid fluids and antibiotics, as well as collection of blood cultures and a serum lactate level. The hour-6 bundle highlights essential elements of continual sepsis care, including repeat assessment of tissue perfusion, repeating a lactate level if initially elevated, and initiation of vasopressors. As more institutions have implemented bundle elements as a systematic method to guide bedside management of sepsis patients, improvements in mortality rates with increased bundle compliance have been observed [3,5,6].

For institutions to receive reimbursement, the Centers for Medicare & Medicaid Services (CMS) echoes the SSC recommendations for sepsis bundle adherence; however, payment is based on compliance with hour-3 and hour-6 bundles. At this time, CMS is not providing reimbursement based on hour-1 compliance. To uphold the standards set forth by CMS and improve sepsis outcomes, our institution launched a team-based “Code Sepsis” protocol in the emergency department in November 2022. Upon Code Sepsis activation, team members, including physicians, advanced practice providers, nurses, and pharmacists, follow an algorithm outlining each of the bundle components to ensure their prompt completion.

Code Sepsis can be activated for any patient with suspected sepsis during their stay in the emergency department. Upon activation, a paper checklist of tasks to be completed is maintained at the bedside for team members to complete based on their specific role. Sepsis providers will be responsible for monitoring the sepsis timer, completing chart documentation related to sepsis care, and overseeing checklist completion. Code Sepsis action items include but are not limited to ongoing clinical assessment at the bedside, ordering and administration of resuscitative fluids and antibiotics, and collection of laboratory studies. The primary purpose of this study was to evaluate the impact of our institution’s protocolized Code Sepsis program on bundle compliance rates. 

Part of the data in this study was previously presented as a meeting abstract and poster at the Society of Critical Care Medicine Annual Congress on February 24, 2025.

## Materials and methods

This retrospective study, conducted at William Beaumont University Hospital, a large academic medical center, included patients aged ≥18 years with severe sepsis or septic shock who received at least one dose of an antimicrobial agent within 24 hours of presentation to the emergency department. Patients admitted between 3/15/22 and 6/15/22 comprised the Pre-Code Sepsis (PC) group, and those admitted between 3/15/23 and 6/15/23 comprised the After-Code Sepsis (AC) group. Patients were excluded if sepsis resuscitative measures began at an outside hospital or if they had an incorrect patient identifier. This study was approved by the institution’s review board.

The AC patient list was obtained from the institution’s sepsis quality assurance committee, which maintains a list of all patients for whom the Code Sepsis protocol was initiated. The PC patient list was identified using International Classification of Diseases, Tenth Revision (ICD-10) codes for sepsis during the prespecified time frame. Patients were included in the PC group via 1:1 matching with the AC group based on admission month until both groups totaled the same number of patients.

Data collection was conducted retrospectively via chart review using the institution’s electronic medical records database. Laboratory values and vitals associated with the presence of systemic inflammatory response syndrome (SIRS) and organ dysfunction were also collected to assess for the presence of severe sepsis or septic shock, as well as culture data collected within the first 48 hours from admission (Table 1) [7].

**Table 1:** Sepsis definitions Reference: American College of Chest Physicians/Society of Critical Care Medicine Consensus Conference. Definitions for sepsis and organ failure, and guidelines for the use of innovative therapies in sepsis aPTT: activated partial thromboplastin time; INR: international normalized ratio; MAP: mean arterial pressure; SBP: systolic blood pressure; SIRS: systemic inflammatory response syndrome

Parameter	Definition*
SIRS	Presence of at least two of the following: temperature >38 °C or <36 °C; heart rate >90 beats/minute; respiratory rate >20 breaths/minute or PaCO_2_ <32 mmHg; white blood cells >12,000/mm^3^ or <4,000/mm^3^
Organ dysfunction	Presence of at least one of the following: SBP <90 mmHg or MAP <65 mmHg; SBP decrease of >40 mmHg from baseline; acute respiratory failure as evidenced by need for invasive or non-invasive mechanical ventilation; serum creatinine >2 mg/dL; total bilirubin >2 mg/dL; platelet count <100,000 bil/L; INR >1.5 or aPTT >60 seconds; lactate >2 mmol/L

The administration date and time of all antimicrobials and fluids within the first 24 hours of admission, as well as steroids within the first 48 hours, were recorded.. Data on all vasopressors utilized throughout the admission, including product type, duration, and vasopressor-free days, were also obtained.

The quality measure set forth by CMS continues to utilize the Severe Sepsis and Septic Shock Early Management Bundle (SEP-1) to evaluate compliance; hence, the SEP-1 definitions of severe sepsis and septic shock were maintained for the purpose of this study [8]. Severe sepsis was defined as the presence of ≥2 SIRS criteria, evidence of organ dysfunction, and documentation of suspected infection within six hours of each other. While formally utilized as a criterion of SIRS, immature bands were not collected, given that complete blood counts with differentials are not routinely ordered on initial admission at the study site. Additionally, while organ dysfunction may also be evidenced by urine output <0.5 mL/kg/hr for two consecutive hours, given inconsistent documentation upon initial presentation, this criterion was not utilized to assess the presence of organ dysfunction. Septic shock was defined as the concern for sepsis and persistent or new-onset hypotension within one hour of completing intravenous crystalloid fluid administration.

Time zero was defined as the earliest time at which all the following were present: ≥2 SIRS criteria, organ dysfunction, and suspicion of infection documented within the patient’s chart. Hour-3 and hour-1 bundles included collection of initial lactate and blood cultures, administration of appropriate broad-spectrum antibiotics, and initiation of 30 mL/kg (using actual body weight) of crystalloid fluid if the patient was hypotensive or presented with septic shock. Fluid volume less than the guideline-directed 30 mL/kg was considered appropriate if a rationale was documented within the patient’s chart. The hour-6 bundle components included measurement of repeat lactate if the initial lactate was elevated, administration of vasopressors if the patient remained hypotensive after fluid resuscitation, and completion of a repeat tissue perfusion assessment if hypotension persisted after fluid administration or if the initial lactate was ≥4 mmol/L.

The primary outcome was hour-3 bundle compliance before and after Code Sepsis implementation. Secondary outcomes included hour-1 and hour-6 bundle compliance. Additional exploratory outcomes included in-hospital mortality and length of stay. Bundle compliance was conducted in an “all-or-nothing” manner, in which all components of each respective bundle needed to be met within the specified time frame after time zero in order to meet compliance.

Antibiotics for each patient were considered appropriate if selection was in accordance with national or institutional guideline recommendations for the suspected source of infection. Furthermore, if more than one antibiotic was needed, the appropriate sequence order of administration must have been as follows: beta-lactams, followed by fluoroquinolones/monobactams, anti-methicillin-resistant *Staphylococcus aureus *(MRSA) agent, and other antimicrobials last. To be deemed an appropriate sequence of administration, the time between the end of the infusion of the first antimicrobial and initiation of the second antimicrobial must have been no longer than 15 minutes in duration.

Statistical analysis was conducted using SPSS Statistics Version 29.0 (IBM Corp., Armonk, NY). Discrete data were analyzed utilizing either Fisher's exact or chi-square statistical analyses, and continuous data were analyzed via 2-sample T-tests (Student’s T-test) or the Mann-Whitney U test. Data are reported either as mean and standard deviation (SD) or median and interquartile range (IQR). A two-tailed p-value of ≤0.05 was considered to be statistically significant. Assuming a 25% increase in hour-3 bundle compliance after code sepsis implementation, it was determined that a total sample size of 98 patients would be needed to detect a difference in the primary outcome with 80% power and an alpha of 0.05. A post-hoc power analysis was conducted to determine the power of the final study population.

## Results

**Figure 1:**
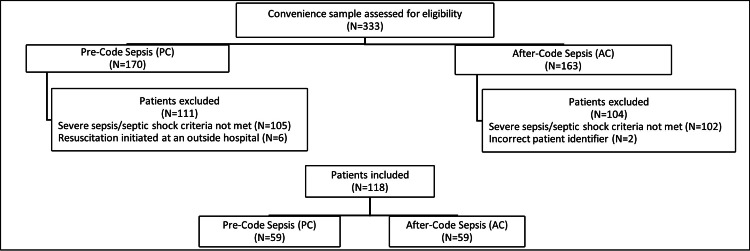
Patient eligibility

Of 730 patients identified, a convenience sample of 333 patients was evaluated for the study (Figure 1).

A total of 118 patients were included for data collection and analysis, with 59 patients in each comparison group. Overall, baseline characteristics were similar between groups, with a mean age of 63 ± 18 years, 45% male, and 42% presenting with septic shock (Table 2).

**Table 2:** Baseline characteristics BMI: body mass index; CKD: chronic kidney disease; COPD: chronic obstructive pulmonary disease; DBP: diastolic blood pressure; ESRD: end-stage renal disease; GCS: Glasgow Coma Scale; HR: heart rate; IQR: interquartile range; MAP: mean arterial pressure; RR: respiratory rate; SBP: systolic blood pressure; SD: standard deviation; WBC: white blood cell

Characteristics	Pre-Code sepsis (n=59)	After-Code sepsis (n=59)	P-value
Demographic data			
Age, years, mean ± SD	64 ± 17	63 ± 19	0.64
Male, n (%)	30 (51)	23 (40)	0.19
Weight, kg, mean ± SD	79 ± 27	75 ± 24	0.43
BMI, kg/m^2^, mean ± SD	28 ± 8	27 ± 9	0.76
GCS, mean ± SD	14 ± 2	14 ± 1	0.35
Past medical history, n (%)			
Hypertension	37 (63)	28 (49)	0.09
Diabetes mellitus	19 (32)	17 (29)	0.68
CKD	14 (24)	14 (24)	0.99
Heart failure	12 (20)	7 (12)	0.21
Active malignancy	11 (19)	11 (19)	0.99
Non-active malignancy	10 (17)	6 (10)	0.28
COPD	6 (10)	4 (7)	0.74
Cirrhosis	4 (7)	2 (3)	0.67
Myocardial Infarction	3 (5)	5 (8)	0.71
ESRD on dialysis	2 (3)	2 (3)	0.99
Social history, n (%)			
Alcohol use	12 (20)	14 (24)	0.65
Tobacco, vaping, or marijuana use	12 (20)	9 (15)	0.47
Intravenous drug use	0 (0)	1 (2)	0.99
Sepsis classification, n (%)			
Septic shock	28 (47)	21 (36)	0.26
Initial vitals and labs			
Highest lactate in 24 h, median (IQR)	3.4 (2.1 – 3.7)	3.5 (2.0 – 3.2)	0.12
Lactate >2 mmol/L, n (%)	46 (78)	42 (71)	0.40
Lactate > 4 mmol/L, n (%)	12 (20)	8 (14)	0.32
Highest WBC/mm^3^, median (IQR)	16.1 (11.3 – 22.8)	14.9 (9.5 – 19.5)	0.21
Lowest WBC/mm^3^, median (IQR)	13.1 (9 – 18)	10.5 (6.9 – 14.9)	0.06
Admission SBP, mmHg, median (IQR)	118 (102 – 137)	120 (105 – 133)	0.96
Admission DBP, mmHg, median (IQR)	66 (56 – 75)	67 (59 – 77)	0.68
Admission MAP, mmHg, median (IQR)	84 (72 – 96)	85 (74 – 94)	0.66
MAP <65 mmHg within 24 h of admission, n (%)	30 (51)	28 (47)	0.71
SBP <90 mmHg within 24h of admission, n (%)	31 (53)	24 (41)	0.20
Admission HR, beats/minute, median (IQR)	111 (97 – 129)	108 (96 – 125)	0.53
Highest HR, beats/minute, median (IQR)	118 (101 – 130)	116 (101 – 132)	0.88
Admission RR, breaths/minute, median (IQR)	20 (19 – 27)	22 (18 – 24)	0.66
Highest RR, breaths/minute, median (IQR)	28 (23 – 34)	29 (24 – 36)	0.20
Initial suspected infection source, n (%)			
Urinary	24 (41)	25 (43)	0.85
Respiratory	17 (29)	23 (39)	0.24
Abdominal	7 (12)	9 (15)	0.59
Skin and soft tissue	6 (10)	5 (8)	0.99
Bone/joint	2 (3)	3 (5)	0.99
Bacteremia	1 (2)	5 (8)	0.20
Unknown	9 (15)	8 (14)	0.79

A trend toward significance was observed for the primary outcome of hour-3 bundle compliance (25% PC vs. 41% AC, p=0.07) (Table 3), with a similar trend for hour-6 bundle compliance (51% PC vs. 64% AC, p=0.13) (Table 4). Hour-1 bundle compliance significantly improved after Code Sepsis implementation (10% PC vs. 24% AC, p=0.03).

**Table 3:** Sepsis hour-3 bundle compliance A p-value <0.05 was considered statistically significant. Note: a different N-value than the overall group is noted in some outcomes based on the number of patients meeting the outcome criteria

Primary outcome	Pre-Code sepsis (n=59)	After-Code sepsis (n=59)	P-value
Hour-3 bundle compliance, n (%)	15 (25)	24 (41)	0.07
Initial lactate obtained	58 (98)	58 (98)	0.99
Blood cultures obtained before antibiotic administration	43 (73)	49 (83)	0.18
Appropriate broad-spectrum antibiotics administered	55 (93)	54 (92)	0.99
Second antibiotic administered within 15 minutes	21 (44) n=48	24 (51) n=47	0.47
Appropriate IV crystalloid bolus initiated if indicated	15 (41) n=37	32 (75) n=32	0.07

**Table 4:** Sepsis hour-1 and hour-6 bundle compliance A p-value <0.05 was considered statistically significant. Note: a different N-value than the overall group is noted in several outcomes based on the number of patients meeting the outcome criteria

Secondary outcomes	Pre-Code sepsis (n=59)	After-Code sepsis (n=59)	P-value
Hour-1 bundle compliance, n (%)	6 (10)	14 (24)	0.03
Initial lactate obtained	52 (88)	57 (97)	0.16
Blood cultures obtained before antibiotic administration	29 (49)	36 (81)	0.19
Appropriate broad-spectrum antibiotics administered	30 (51)	35 (59)	0.35
Appropriate IV crystalloid bolus initiated if indicated	15 (42) n=36	27 (82) n=33	0.001
Hour-6 bundle compliance, n (%)	30 (51)	38 (64)	0.13
Repeat lactate level obtained	39 (85) n=46	44 (98) n=45	0.31
Documentation of tissue perfusion assessment	2 (7) n=28	15 (60) n=25	<0.001
Vasopressors initiated	13 (59) n=22	7 (54) n=17	0.76

Hospital length of stay was significantly shorter in the AC group (8.1 (4.8-12.9) PC vs. 5.1 (2.9-8.2) AC, p=0.009). No significant difference between all-cause mortality (20% PC vs. 10% AC, p=0.12) or hospice disposition (8% PC vs. 2% AC, p=0.20) was noted between groups; a significantly higher number of patients were discharged home in the AC group (56% PC vs. 73% AC, p=0.05) (Table 5).

**Table 5:** Hospital length of stay, disposition, and mortality ^*^Length of stay in surviving patients Note: a different N-value than the overall group is noted in several outcomes based on the number of patients meeting the outcome criteria. A p-value <0.05 was considered statistically significant IQR: interquartile range

Exploratory outcome	Pre-Code sepsis (n=59)	After-Code sepsis (n=59)	P-value
Length of stay^*^, median (IQR)			
Overall hospital length of stay, days	8.1 (4.8 – 12.9) n=47	5.1 (2.9 – 8.2) n=53	0.009
Emergency department length of stay, hours	6.5 (5.3 – 9.3) n=47	7.5 (6.1 – 10.0) n=53	0.05
Intensive care unit length of stay, days	4.0 (2.4 – 6.1) n=13	2.8 (1.3 – 23.3) n=7	0.90
Disposition, n (%)			
Home	33 (56)	43 (73)	0.05
Long-term care facility	7 (12)	6 (10)	0.76
Rehabilitation center	2 (3)	3 (5)	0.64
Hospice	5 (8)	1 (2)	0.20
In-hospital all-cause mortality – n (%)	12 (20)	6 (10)	0.12

**Figure 2:**
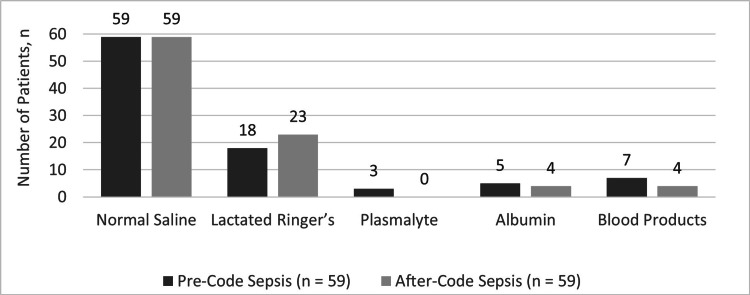
Fluid selection - first 24 hours from time zero of suspected sepsis

Regarding fluid resuscitation, a significantly higher proportion of patients in the AC group received an appropriate initial crystalloid bolus within the hour-1 bundle (42% PC vs. 82% AC, p=0.001), and a trend towards improved compliance was also observed in the hour-3 bundle (41% PC vs. 75% AC, p=0.07) (Tables 3, 4). All patients received normal saline as part of their fluid resuscitation, with the next most common fluid utilized being Lactated Ringer’s (31% PC vs. 40% AC, p=0.33) (Figure 2). Total fluid volume (4109 ± 1812 PC vs. 3942 ± 1633 AC, ml, p=0.60) as well as total weight-based fluid volume (56 ± 28 PC vs. 57 ± 27 AC, ml/kg (actual body weight), p=0.74) within the first 24 hours were similar between groups.

The majority of patients were noted to have received appropriate broad-spectrum antibiotics by hour-3 (93% PC vs. 92% AC, p=0.99) (Table 3). The most common antimicrobial agents utilized included cefepime (41% PC vs. 42% AC, p=0.85, piperacillin/tazobactam (46% PC vs. 36% AC, p=0.26), and vancomycin (69% PC vs. 56% AC, p=0.18). Utilization of ceftriaxone significantly increased after Code Sepsis implementation (15% PC vs. 36% AC, p=0.01) while the use of meropenem significantly decreased (15% PC vs. 2% AC, p=0.02).

Cumulative duration of vasopressor therapy was significantly lower after Code Sepsis implementation (69 (26-170) PC vs. 14 (10-24) AC, hours, p=0.01) (Table 6). Norepinephrine followed by vasopressin were the most commonly utilized vasopressors during admission (Figure 2). Additionally, the use of steroids for the first 48 hours was similar between groups.

**Table 6:** Vasopressor and steroid use ^*^Cumulative vasopressor therapy duration included only patients who received vasopressors during admission. ^**^Vasopressor-free days calculated as the number of consecutive days free of vasopressor support up to 28 days. ^***^Patients were counted for steroid use if on hydrocortisone 50 mg every six hours or 100 mg every eight hours, dexamethasone ≥6 mg, or methylprednisolone ≥40 mg IQR: interquartile range

Vasopressor utilization during admission	Pre-Code sepsis (n=59)	After-Code sepsis (n=59)	P-value
Total number of patients requiring vasopressor therapy, n (%)	16 (27)	8 (14)	0.06
Cumulative vasopressor therapy duration^*^, hours, median (IQR)	69 (26 – 170)	14 (10 – 24)	0.01
Vasopressor-free days^**^, mean ± SD	26 ± 7	27 ± 6	0.40
Steroid utilization for 48 hours after admission^***^			
Hydrocortisone, n (%)	9 (15)	6 (10)	0.40
Methylprednisolone, n (%)	2 (3)	4 (7)	0.67
Dexamethasone, n (%)	2 (3)	2 (3)	0.99

**Figure 3:**
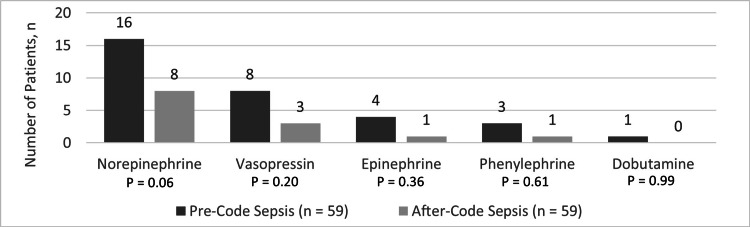
Vasopressors utilized

## Discussion

While sepsis bundles continue to be promulgated by the Surviving Sepsis Campaign, and many medical institutions have adopted protocols to enhance bundle completion, robust evidence defining the true clinical impact of bundle compliance remains limited. Consequently, hesitation to implement sepsis protocols continues due to concerns of an undetermined degree of clinical benefit at the potential cost of propagating antibiotic overuse and possible implementation challenges. The feasibility of hour-3 compliance has been observed since the original publication of the hour-3 bundle in the 2012 SSC guideline update; however, data on mortality impact are mixed, with some publications observing reduced mortality with hour-3 bundle compliance, whereas other publications have not observed this benefit [9-11]. This study sought to explore the impact of a protocolized approach to sepsis management on bundle compliance and the potential effect on clinical outcomes.

Code Sepsis implementation led to an overall trend toward improved hour-3 bundle compliance, achieving a total of 41% compliance rate after implementation. While this percentage is lower than those observed after protocol implementation in prior literature, such as an 82.5% hour-3 compliance reported by Seymour et al. [5] and 63.3% by Venkatesh et al. [12], the overall increase from 25% to 41% compliance in this study reflects the degree of improvement also observed in the aforementioned article. Furthermore, Lynn et al. observed trends in monthly hour-3 compliance across a five-year study period, reporting improved compliance over time as well as a 14.5% lower mortality rate for those meeting compliance, indicating that the longer the sepsis program was instated, the better the compliance became [10]. It is thus encouraging to note that over the span of eight months from Code Sepsis conception to the end of the study time frame at this institution, a 60% increase in the hour-3 compliance rate has been observed and is similarly expected to increase over time.

In this study, improved compliance rates were most notably demonstrated within the hour-1 bundle. Given that the recommendation for the hour-1 bundle is new to SCC guideline recommendations since 2018, there is less exploration of the feasibility and benefit of this bundle as compared to the outcomes associated with hour-3 and hour-6 bundles [13]. Prior evaluations of hour-1 bundle compliance have demonstrated no impact on 28-day mortality or in-hospital mortality [14,15]. However, Venkatesh et al. found significantly reduced ICU admission rates in patients who met hour-1 and hour-3 bundle compliance, as well as no observed adverse impact on antimicrobial utilization [12]. The positive findings of hour-1 compliance in this study indicate its feasibility; however, further exploration of its clinical impact is warranted.

Regarding significantly improved utilization of an appropriate fluid bolus within the first hour, similar rates of common comorbidities precluding the use of a 30 mL/kg bolus, such as end-stage renal disease, heart failure, or severe aortic stenosis, were seen between both groups. Consequently, the rates of providers deciding to utilize less than the 30 mL/kg volume due to such reasons would be expected to be similar between groups, indicating a low likelihood of these comorbidities skewing fluid compliance results. Overall, the improvement in appropriate fluid resuscitation in the AC group demonstrated that Code Sepsis implementation led to greater guideline-concordant sepsis treatment [1]. The improvement was driven both by increased provider utilization of an appropriate initial fluid bolus as well as increased documentation of the rationale for giving less than 30 ml/kg fluid volume. Even so, the precise benefit behind the use of a 30 mL/kg crystalloid for initial fluid resuscitation has not been strongly defined in the literature.

Optimal resuscitation volume has been widely studied since Rivers et al. reported reduced mortality with early goal-directed therapy in sepsis patients initially resuscitated with 20 - 30 mL/kg crystalloid fluid [16]. Other studies have seen varied results. For example, delayed completion of the initial fluid bolus between 6-12 hours after suspected sepsis was not associated with increased in-hospital mortality as compared to completion within six hours in the study by Seymour et al. (odds ratio, 1.01 per hour; 95% CI, 0.99 - 1.02; p=0.21) [5]. Conversely, a more recent study evaluated mortality in patients who received the guideline-directed ≥30 mL/kg fluid in the first six hours of sepsis as compared to those who received less volume and observed lower risk of in-hospital and 30-day mortality in the ≥30 mL/kg fluid group (odds ratio, 0.80; 95% CI, 0.66 - 0.96; p<0.05) [17]. While the degree of benefit remains unclear, with current guideline recommendations and existing level of data, it remains beneficial for our institution to aim for the timely completion of a 30 mL/kg bolus unless the risk of fluid overload clearly outweighs the benefit of this resuscitative volume.

For the individual hour-1 bundle component of antibiotic administration, a possible reason for less observed improvement in compliance within this bundle could include the inherent clinical tendency to allow more time for assessment of patients with less clinically obvious signs of sepsis before initiating antibiotics. The 2016 SSC guidelines' emphasis on antibiotic administration to all sepsis patients within one hour, irrespective of the presence or absence of septic shock, has been subject to scrutiny, most notably by the Infectious Diseases Society of America (IDSA) on the basis of these recommendations propagating antibiotic overuse [18,19]. Consequently, the most recent SSC 2021 guidelines provide different recommendations for antimicrobial timing based on the presence of septic shock as well as the degree of certainty of a sepsis diagnosis [1].

Based on these guidelines, for patients with less clinically overt sepsis diagnosis, it may be appropriate to adopt a “watchful waiting” approach and to administer antimicrobials within three hours if suspicion of sepsis continues, rather than targeting administration within one hour for all patients. At this institution, clinicians may inherently employ a more conservative approach to antimicrobial administration to avoid antibiotic overuse, irrespective of Code Sepsis being initiated. This may also be reflected by the significantly reduced use of meropenem and increased utilization of ceftriaxone in the initial phases of sepsis treatment in the AC group. Over half of the patients in both groups received the appropriate antibiotics within the hour-1 bundle, which is similar to the rates of patients with septic shock seen in the study population, suggesting that prompt antimicrobial administration may still be prioritized in this population subset that would have the highest benefit. Overall, the majority of patients ultimately received the appropriate antimicrobial within the hour-3 bundle, which reflects current guideline recommendations.

In terms of vasopressor utilization, this institution’s preference for norepinephrine with the addition of vasopressin as needed reflects guideline recommendations for initial vasopressor management in sepsis-induced hypoperfusion or septic shock [1]. Interestingly, the total vasopressor duration was found to be significantly lower in the AC group despite similar rates in patients being indicated for vasopressors between groups and similar utilization of steroids in the initial 48-hour period after time zero. Possible reasons for this difference could be that higher rates of appropriate fluid boluses utilized in the AC group contributed to less overall vasopressor requirements. Additionally, a slightly lower percentage of septic shock patients was found in the AC group, although this was not statistically significant (Table 1). Patients in the PC group may also have had subsequent indications for which vasopressor therapy was continued. Ultimately, vasopressor-free days were comparable between groups, indicating that patients had similar hemodynamic outcomes long-term. While this study did not see a significant difference in mortality between groups, significantly shorter overall length of stay after Code Sepsis implementation is noteworthy, given that sepsis is known to lead to substantial costs to the healthcare system [20].

All centers aim for full compliance with the hour-3 bundle and the hour-6 bundle approach to sepsis care based on CMS requirements. One strength of this study is the proactive approach in the identification post implementation of sepsis patients upon first presentation, as it represents a real-world experience. The methods of the study were compared before versus after implementation of the Code Sepsis approach, which evaluates the impact of education and awareness of this population of patients. Another strength of the study is the feasibility of the practice of the hour-1 bundle compliance since hour-1 bundle compliance is a newer SSC recommendation with a paucity of data defining its clinical impact. Second, the study highlights the complexity of bedside care as definitions of sepsis, clinical outcomes, and reimbursement qualifications continue to evolve. Our study suggests that utilization of a sepsis protocol can equip clinicians with more streamlined management of sepsis at the bedside while allowing physicians to individualize therapy to maximize delivery of patient-centered care. In addition, the study evaluated more than 98 patients, which was the sample size needed for adequate powering of the primary outcome.

This study has several limitations. Primarily, the single-center design with a smaller population may limit the generalizability of the results. In light of the retrospective design, selection bias may have occurred, although we attempted to account for this type of bias with case matching. Inherently, retrospectively designed studies are limited in that data may be missing or incorrectly charted within the electronic health records. The generalizability of this study to inpatient populations due to nursing staffing models and antibiotic and fluid availability may vary since the Code Sepsis protocol was implemented only within the emergency department. Furthermore, utilization of older definitions of severe sepsis from the original 2004 SSC guidelines may pose difficulty with extrapolating findings to current sepsis populations; however, when comparing the criteria for severe sepsis or septic shock to the most current sepsis definitions, over half of the patient population in this study would be characterized as having sepsis-induced hypoperfusion or septic shock within the most recent SSC guidelines [1].

As such, this study represents a fairly homogenous mixture of all sepsis severities that would be commonly seen in practice. Mortality rates remain ambiguous in current sepsis literature as data have remained largely retrospective, and, if observed prospectively, are underpowered to detect mortality differences when evaluating overall bundle compliance. Hence, future study directions could include the exploration of the true impact of sepsis bundles on mortality.

## Conclusions

This study demonstrated that implementation of a protocolized Code Sepsis program was associated with a trend toward improved compliance in the hour-3 and hour-6 bundles, as well as significantly improved hour-1 sepsis bundle compliance and reduced hospital length of stay. A protocolized approach to sepsis management has been shown to enhance bundle compliance not only in the emergency department but also within acute care inpatient settings. Given the significant impact sepsis has on mortality and morbidity, further adequately powered prospective studies are required to analyze the impact of bundle compliance on the reduction of mortality and morbidity.
